# Unusual spin dynamics in topological insulators

**DOI:** 10.1038/srep14844

**Published:** 2015-10-06

**Authors:** Balázs Dóra, Ferenc Simon

**Affiliations:** 1 BME-MTA Exotic Quantum Phases Research Group, Budapest University of Technology and Economics, PoBox 91, H-1521 Budapest, Hungary; 2 Department of Physics, Budapest University of Technology and Economics and MTA-BME Lendulet Spintronics Research Group (PROSPIN), PoBox 91, H-1521 Budapest, Hungary

## Abstract

The dynamic spin susceptibility (DSS) has a ubiquitous Lorentzian form around the Zeeman energy in conventional materials with weak spin orbit coupling, whose spectral width characterizes the spin relaxation rate. We show that DSS has an unusual non-Lorentzian form in topological insulators, which are characterized by strong SOC, and the anisotropy of the DSS reveals the orientation of the underlying spin texture of topological states. At zero temperature, the high frequency part of DSS is universal and increases in certain directions as *ω*^*d*−1^ with *d* = 2 and 3 for surface states and Weyl semimetals, respectively, while for helical edge states, the interactions renormalize the exponent as *d* = 2*K* − 1 with *K* the Luttinger-liquid parameter. As a result, spin relaxation rate cannot be deduced from the DSS in contrast to the case of usual metals, which follows from the strongly entangled spin and charge degrees of freedom in these systems.

Strong correlation effects manifest as unusual behavior of physical response functions. Of these, the frequency and momentum dependent spin susceptibility, *χ*(*q*, *ω*), played a pivotal role in the study of e.g. high-temperature superconductors^1^, spin-ice compounds^2^, and the fundamental description of magnetic resonance experiments in correlated systems^3^. This response function is available experimentally using *ac* magnetization measurements, neutron scattering, magnetic resonance, Mössbauer spectroscopy, spin-resolved STM, or microwave cavity perturbation experiments. Common to these method is that it is difficult to deduce the full *ω* dependent signal, the analysis of experiment therefore relies on the theoretical description of the susceptibility.

The long wavelength spin susceptibility, *χ*(*q* → 0, *ω*), called the *ac* or dynamic spin susceptibility (DSS), indicates dissipative processes and remains in the focus of interest when studying the nature of correlations in emergent materials, such as e.g. those manifesting the spin-liquid phase^4^. DSS is also important in identifying the transition temperature of spin-glasses^5^ and superconductors^6^, characterizing superparamagnetism of small ferromagnetic nanoparticles^7^, or examining the nature of magnetic phase transitions. Another highly relevant reason to study DSS is that it provides a measure of spin-relaxation rate, whose knowledge is in turn important for spintronics applications^8^. DSS is characterized in the usual materials (where spin-orbit interaction is small) by a Lorentzian^9^,^10^,^11^,^12^, which is peaked at the Zeeman energy and whose linewidth provides a direct measure of the spin-relaxation rate.

In a normal metal without spin orbit coupling (SOC), the DSS reduces to *ωδ*(*ω* ± *B*) with *B* the Zeeman field, even in the presence of non-magnetic impurities and electron-electron interaction, which yield a finite lifetime for the electrons, since these preserve the rotational invariance of spin space, and cannot induce a finite spin lifetime on their own. The essential ingredient for spin relaxation is the breaking of this symmetry, which is naturally provided by the SOC. Then, combined with the above sources of relaxation, the sharp Dirac-delta peak broadens and in many cases, assumes a Lorentzian form, whose width is determined by the SOC and the momentum lifetime. This usually occurs in the case of weak spin orbit coupling (SOC).

However, SOC is usually the dominant energy scale in topological insulators^13^,^14^ which strongly entangles their magnetic properties with their charge response, and causes their peculiar helical spin structure^13^. While existence of topological states can be revealed by imaging their band structure via ARPES^13^ or by transport measurement^15^, the detection of the underlying spin texture^13^,^14^, resulting from the conspiracy of spin and charge degrees of freedom, represents a challenging task. The DSS is uniquely sensitive to the spin arrangement and an unusual, non-Lorentzian behavior of the DSS might occur. Here, we study DSS in topological insulators in the full temperature, doping, Zeeman energy and frequency range. We do find a non-Lorentzian form of the DSS and most surprisingly a non-zero value of the DSS even in the large frequency limit. This, on the one hand, is identified as a new hallmark of time reversal symmetry protected^16^ topological insulators in various dimensions, stemming from their unique spin texture, as we show below for i) the spin Hall edge state, ii) its strongly correlated counterpart, the helical liquid in 1D, iii) 2D helical Dirac fermions, iv) and the Weyl semimetal in 3D. We stress that the contribution from bulk states is neglected and our results on the boundary modes are valid for energies below the bulk gap of topological insulators. On the other hand, this also implies that the spin-relaxation rate cannot be determined from the DSS, much as its knowledge is desired for prospective spintronics applications. This result is understood in analogy to the case of optical conductivity of *neutral* graphene: it does not follow the usual Drude-Lorentz form due to two-band excitations, therefore it cannot be used to determine the momentum relaxation rate^17^.

## 1D Dirac Hamiltonian: The Spin-Hall Edge State

We consider the spin-filtered edge states of a quantum spin-Hall insulator^15^,^18^,^19^, whose effective Hamiltonian is


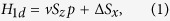


where *v* is the Fermi velocity, *S*_*i*_ with *i* = *x*, *y*, *z* is the spin operator of the electron and *p* is the 1D momentum along the edge. The energy spectrum is 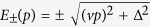 and Δ ~ *B* is the Zeeman term from a static magnetic field *B*, which opens a gap in the spectrum.

The DSS requires the calculation of the spin response function, which reads in the time domain as


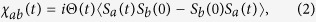


where *a*, *b* = *x*, *y*, or *z* and *S*_*a*_(*t*) = exp(−*iH*_1*d*_*t*)*S*_*a*_exp(*iH*_1*d*_*t*) can be calculated using the matrix structure of *H*_1*d*_, similarly to ref. 20. A given momentum plays the role of an effective magnetic field, which acts on the physical spin. Therefore, the knowledge of the *χ*_*ab*_(*t*) correlator yields directly the DSS. Using the eigenfunctions of Eq. (1), the time dependent correlation function for a given momentum *p* is calculated, yielding the imaginary part of the DSS at half filling and *T* = 0 after Fourier transformation as


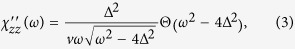



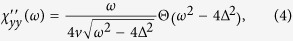



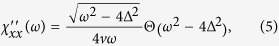



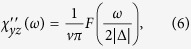


where 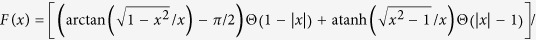 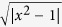. The DSS is thus strongly anisotropic, contains an off-diagonal term and deviates from the ideal Lorentzian form. Depending on the component of DSS (i.e. *χ*_*ab*_), the DSS diverges or vanishes at the gap edge (Δ) and approaches a finite constant value or vanishes with increasing frequency.

In the Δ = 0 limit, *S*_*z*_ is conserved ([*S*_*z*_, *H*_1*d*_] = 0), therefore 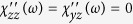, while 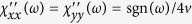, which is the typical density of states in 1D. The anisotropy of the DSS, namely that the components (*χ*_*xx*_ and *χ*_*yy*_) perpendicular to the spin orientation *z* overwhelm *χ*_*zz*_, follows from the helical structure of the edge state.

The electric current operator is given by *j*_*x*_ = *evS*_*z*_, therefore the optical conductivity of the spin-Hall edge state measure directly 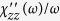. Note that the other components of DSS are not accessible by optical means. Additionally, a finite *ac electric* current can be induced along the edge in the presence of an *ac magnetic* field in the *y* direction due to the finite value of *χ*_*yz*_(*ω*), as a manifestation of the magnetoelectric effect^13^,^14^. In particular, Re 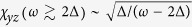.

The results for the diagonal susceptibilities can be extended to finite doping and temperature by multiplying the calculated *χ*_*aa*_’s by 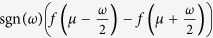 (except for the case of the helical liquid), where *f*(*E*) = 1/(exp(*E*/*k*_*B*_*T*) + 1) with *T* the temperature and *μ* the chemical potential. At *T* = 0, a finite chemical potential introduces an additional gap of 2|*μ*|, and leaves the rest intact. At high temperature, it gives a |*ω*|/4*T* multiplicative factor to the susceptibilities.

In the presence of disorder, the sharp features in the DSS will be rounded and smoothened, such as the square root singularities in Eqs. 3-5, 15, 15, but its high frequency part is not expected to be influenced by disorder (which was essential for normal metals to get a finite spin lifetime), similarly to how the flat optical conductivity of graphene is insensitive to disorder^17^. Therefore, the resulting lineshape is still far from being a Lorentizian and the overall shape of the DSS is still given by its disorder free form.

A finite perpendicular magnetic field Δ opens up a gap in the spectrum, and the resulting state becomes immune with respect to interactions as long as |*μ*| ≪ Δ. In the absence of the gap, the density of states is finite for arbitrary chemical potential, and the interactions profoundly alter the low energy excitations, as is customary in 1D^21^. The results obtained below apply also in the case of a finite gap, unless *μ* ~ Δ ~ *ω*.

## Helical Liquid

The helical edge state of the spin-Hall insulator forms a strongly correlated system, i.e. a helical liquid, when electron-electron interaction is taken into account, resembling to a spinless Luttinger liquid (LL)^22^,^23^,^24^,^25^. The Hamiltonian in Eq. (1) is rewritten in second quantized form as^26^


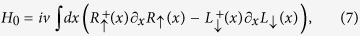


which is a peculiar half of a spinful LL, lacking the *R*_↓_ and *L*_↑_ operators. Here, *R*_↑_(*x*)/*L*_↓_(*x*) annihilates a right/left-moving electron at position *x* with spin ↑/↓.

The time reversal invariant electron-electron interaction consists of the chiral (*g*_4_) and the forward scattering (*g*_2_) terms,


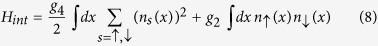


with 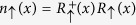 and 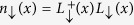. These interactions give rise to Luttinger liquid behaviour^22^,^23^,^24^ with LL parameter *K* and renormalized velocity *v*_*F*_, and *K* = 1 and *v*_*F*_ = *v* in the non-interacting limit. The bosonized Hamiltonian reads as


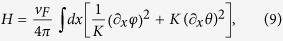


with the dual fields *θ* and *φ*, satisfying 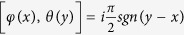.

**Figure 1:**
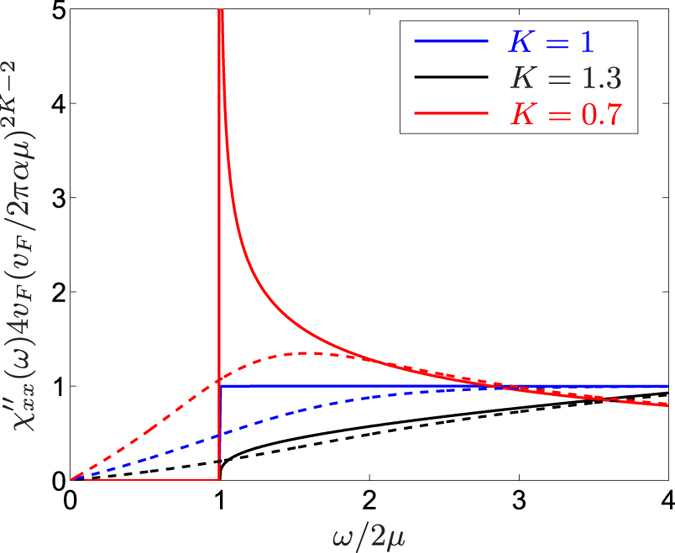
The dynamical spin susceptibility of the helical liquid is shown for *T* = 0 (solid lines) and *T* = *μ*/2 (dashed lines) for several values of the LL parameter.

The DSS of the helical liquid is evaluated similarly to the 2*k*_*F*_ *charge* susceptibility of a spinless LL^21^. The spin flip operator is translated to the bosonic language as 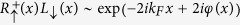. In the absence of perpendicular magnetic field, we obtain 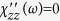 and 
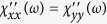
 as


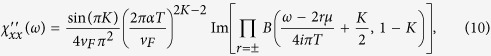


where *B*(*x*, *y*) = Γ(*x*)Γ(*y*)/Γ(*x* + *y*) is the Euler integral of the first kind with Γ(*x*) being the Euler’s integral of the second kind^27^, *α* is a short distance regulator and *v*_*F*_/*α* represent a high energy cutoff and is shown in Fig. 1 for some representative cases. At *T* = 0, Eq. (10) exhibits the typical power law correlation function of a LL as


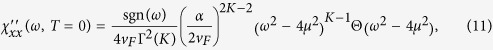


while in the high temperature limit with *T* ≫ *ω*, *μ*, it yields


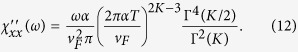


**Figure 2:**
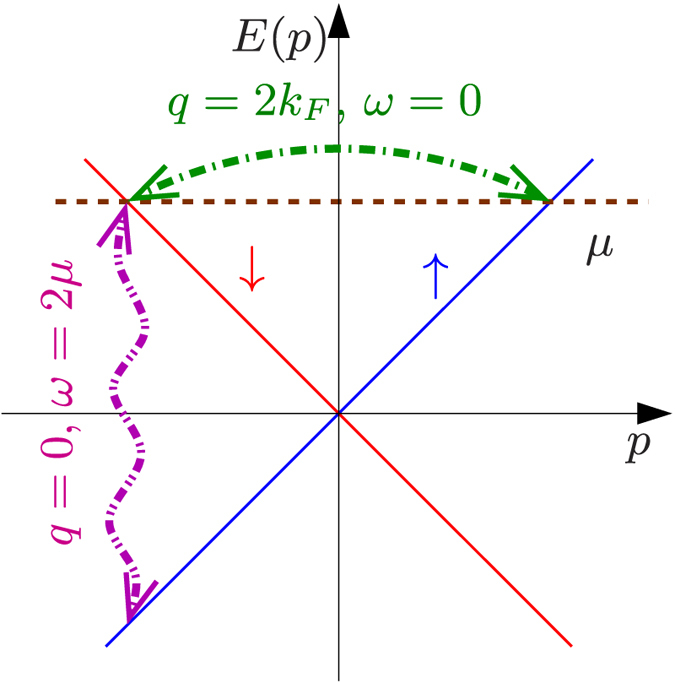
The two possible spin-flip processes in the helical liquid, the blue (up spin) and red (down spin) lines denote the bare, spin filtered dispersion. The *q* = 0 process, corresponding to the vertical magenta line, is absent in a normal LL and requires a finite frequency threshold 2*μ*, while the green arrow denotes a gapless, *q* = 2*k*_*F*_ momentum transfer process, which does not contribute to DSS, except for *μ* = 0, when these two processes coincide.

In spite of the formal similarity to the 2*k*_*F*_, finite frequency response of normal LLs, Eq. (10) describes a completely different physical process, which usually involves high energy transfer and is beyond the realm of the LL paradigm. While the former is gapless in *ω* and accounts for a “horizontal” interband process with 2*k*_*F*_ momentum transfer, the latter stems from a *q* = 0 “vertical” interband transition and is gapped at *T* = 0 with the threshold frequency of interband transition 2*μ*, as shown in Fig. 2. Only at *μ* = 0, these two processes become identical. By the replacement 2*μ* → 2*μ* ± *vq* in Eq. (10), the full wavevector dependence of the dynamical susceptibility is obtained. This indicates that the chemical potential dependent DSS is equivalent to measure the full wavevector dependent susceptibility, accessible by e.g. neutron scattering. The charge response of helical liquids^28^ also features interaction effects, which differ from the spin response.

The effect of the Zeeman term can be taken into account qualitatively following ref. 29. Similarly to the non-interacting case, a gap opens in the spectrum immediately, which scales as Δ ~ |*B*|^1/(2−*K*)^, and reproduces the noninteracting, Δ ~ |*B*| relation for *K* = 1. This completely suppresses the spin response for *ω* < 2Δ, while for *ω* ≫ 2Δ, the previous results are recovered.

**Figure 3:**
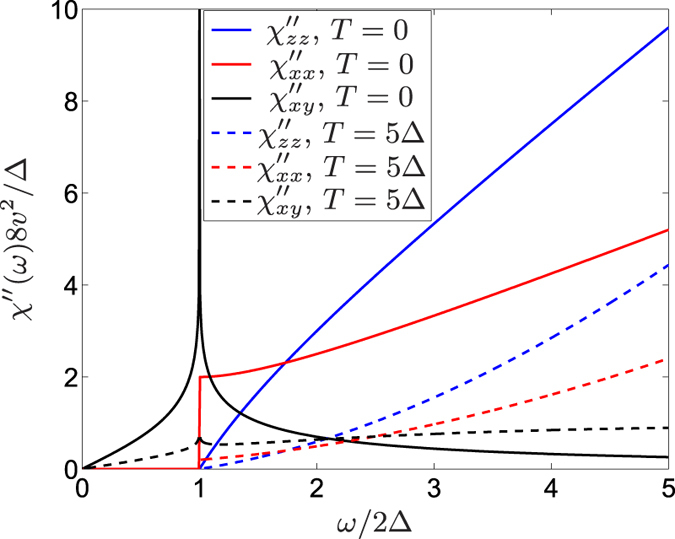
The dynamical spin susceptibility of the 2D topological surface state is shown for *T* = 0 (solid lines) and *T* = 5Δ (dashed lines) at half filling. For comparison with the other susceptibilities, 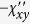 is plotted.

This very broad spin response is reminiscent of that in the XXZ Heisenberg model^21^, which describes frozen charge degrees of freedom due to the strong on site repulsion between electrons. The helical liquid, on the other hand, operates in the opposite, weakly interacting itinerant electron limit, but the strong SOC entangles the spin excitations with the charge degrees of freedom, resulting in a broad signal.(Fig. 3).

In particular, a strongly repulsive helical liquid with *K* ≪ 1 produces significantly larger spin responses as opposed to its weakly or attractively interacting counterpart: the (2*παT*/*v*_*F*_)^2*K*−2^ factor significantly enhances/suppresses the spin susceptibility in the repulsive (*K* < 1)/attractive (*K* > 1) case. For *K* = 1, our previous expressions for the non-interacting case are recovered.

Eq. (10) is to be contrasted to the spin response of a spinful LL, which in the presence of SU(2) invariant interactions, reduces to *ωδ*(*ω* ± *B*) with *B* the Zeeman field, in spite of the fractionalization of the original fermionic excitations into new type of collective bosonic modes. Departures from this highly idealized limit imply the inclusion of various SOC terms into the LL Hamiltonian^30^,^31^ as a weak perturbation on the band structure. Our starting point, on the other hand, is the completely opposite situation, when the SOC determines and dominates the band structure, therefore the SU(2) spin rotational symmetry is severely broken and cannot be considered as a weak perturbation.

## 2D Dirac Hamiltonian

By increasing the dimensionality, the surface states of 3D topological insulators is described by the familiar Dirac equation^13^, given by


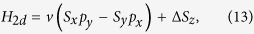


where Δ is a mass gap, stemming from a thin ferromagnetic film covering the surface of TI or by a perpendicular magnetic field. The eigenenergies are 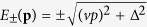. The electromagnetic response of these surface states was considered in refs 32,33.

The time dependent correlation function is obtained similarly to the 1D case, and the DSS at *T* = 0 and half filling is


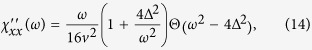



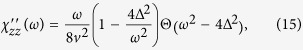



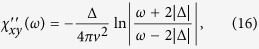


and 
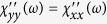
. Note that 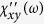 is responsible to the “half quantum Hall effect”, i.e. the *e*^2^/2*h* Hall conductivity in topological insulators^13^. Since the electric current operator is related to the spin due to the strong SOC, the in-plane optical conductivity satisfies 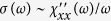. Interestingly, this also agrees with the *charge* response, i.e. the interband contribution to the optical conductivity of (gapped) monolayer graphene^34^. While 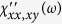 is measurable by optical means as well, the *zz* component can only be probed by magnetic susceptibility measurements. In the Δ = 0 limit, the relation 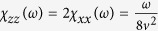 holds where the last expression is the typical density of states of e.g. graphene^35^. The factor 2 follows from the spin structure of Eq. (13): *S*_*z*_ sees two perpendicular spin components (*x* and *y*), which contribute to the response, while an in plane component feels only the other in-plane component but not *S*_*z*_. Similarly to the 1D case, the anisotropy of DSS indicates the orientation of the spin texture. Qualitatively similar diagonal susceptibilities were derived numerically in ref. 36 for finite size topological insulator nanoribbons, while our calculations apply in the thermodynamical limit.

The effect of a short range electron-electron interaction (e.g. Hubbard model) is practically negligible here, as it is termed irrelevant in the renormalization group sense and can only renormalize the band parameters in the weak coupling limit. Additional terms in the Hamiltonian (hexagonal warping^37^, Rashba spin-orbit coupling^14^) can also be present but these can be neglected close to the band touching point (within 50 meV for the warping term).

These non-Lorentzian lineshapes are expected to be robust in the presence of disorder, similarly to the optical conductivity of graphene, which becomes slightly rounded at the gap edge^34^, set by the Zeeman term, but the gross features are well described by the calculations in the clean case. The experimental data on the optical conductivity of graphene^17^ also agrees with calculations performed in the clean limit.

## Weyl Semimetal

Inspired by the exciting physics of graphene and topological insulators, nodal semimetals in 3D are currently under investigation^38^,^39^,^40^. The Weyl Hamiltonian exhausts all three spin operators as


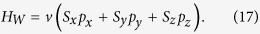


This Hamiltonian is valid below a high energy cutoff, related to the bandwidth, similarly to the Dirac equation description of graphene^35^. The Zeeman energy simply shifts the position of the zero energy state in the momentum space and does not open a gap in the above Hamiltonian. The DSS follows from Eqs. (14-15), 30, after replacing Δ with *k*_*z*_ and performing the *k*_*z*_ integral, becoming isotropic and diagonal as


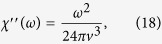


being proportional to the density of states of Weyl semimetals. The isotropic spin response follows from the isotropic, hedgehog like spin texture around the Weyl node in Eq. (17). Impurity scattering is an irrelevant perturbation for the present case and does not change the leading frequency dependence of the DSS at low temperatures, similarly to the optical response of Weyl semimetals^41^. Similarly to our lower dimensional analysis, the optical conductivity follows as 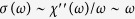 as in ref. 41.

## Detection

Experimentally, the DSS is directly measured by the electron spin resonance (ESR) method, whose signal intensity is^12^ 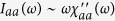. Usually, the conventional ESR method together with the nuclear magnetic resonance (NMR) in solid state systems has limited importance in 2D and especially 1D due to the small number of available states (small density of states compared to 3D), which results in weak signals. Nevertheless, by considering an ensemble of 1D nanowires and crystals, the ESR signal can possibly be detected similarly to the NMR spectra^42^ of related materials. Additionally, one can also use the recently proposed source-probe setup to measure the DSS^43^. The DSS is accessible in a cold atomic realization of these states (see e.g. ref. 44), featuring also the tunability of the interaction strength by standard techniques^45^, by measuring the spin-sensitive Bragg signal, yielding the spin-structure factor.

## Conclusions

We have investigated the dynamic spin susceptibility in topological insulators and Weyl semimetals. The DSS exhibits a non-Lorentzian form of the DSS and a non-zero value even in the large frequency limit. This we identify as a new hallmark of time reversal symmetry protected topological insulators, which originates from their unique spin texture.

## Additional Information

**How to cite this article**: Dóra, B. and Simon, F. Unusual spin dynamics in topological insulators. *Sci. Rep.* **5**, 14844; doi: 10.1038/srep14844 (2015).
